# Provision of Ubiquitous Tourist Information in Public Transport Networks

**DOI:** 10.3390/s120911451

**Published:** 2012-08-24

**Authors:** Carmelo R. García, Ricardo Pérez, Francisco Alayón, Alexis Quesada-Arencibia, Gabino Padrón

**Affiliations:** Institute for Cybernetics, University of Las Palmas de Gran Canaria, Campus de Tafira, Las Palmas de Gran Canaria, Las Palmas 35017, Spain; E-Mails: rperez@dis.ulpgc.es (R.P.); falayon@dis.ulpgc.es (F.A.); aquesada@dis.ulpgc.es (A.Q.-A.); gpadron@dis.ulpgc.es (G.P.)

**Keywords:** ubiquitous computing frameworks, service-orientation, intelligent transport systems

## Abstract

This paper outlines an information system for tourists using collective public transport based on mobile devices with limited computation and wireless connection capacities. In this system, the mobile device collaborates with the vehicle infrastructure in order to provide the user with multimedia (visual and audio) information about his/her trip. The information delivered, adapted to the user preferences, is synchronized with the passage of vehicles through points of interest along the route, for example: bus stops, tourist sights, public service centres, *etc.*

## 1. Introduction

The availability of user-adapted information is an essential aspect of public transport systems, since it helps to improve the quality of service and constitutes a basic resource in complex and dynamic transport systems. According to the studies conducted by different local, regional, national and even international organizations, such as the University of Michigan Transport Research Institute [1] and the European Conference of Ministers of Transport (ECMT) [2], the information should be adapted as needs vary according to the type of person using the service. Indeed, for the information to be effective for the traveller, it must be comprehensible and coherent. Thus it is vital to know what travellers really need; this knowledge involves identifying the different groups of travellers and their specific requirements. For people with specific needs, accessing a public transport system can be a source of concern, from the moment they access the service, because of the physical difficulties experienced in accessing the stations or vehicles, or the uncertainty of unexpected situations during the trip. Following Stradling [3], these feelings discourage them from using the public transport.

The main aim of the system presented in this paper is to offer suitable information during the trip to the users of a public transport service, adapting the information to the special requirements of users, for example visual information and physical warning (vibrations or sounds) for deaf or blind people, respectively. Text information in various languages for tourists or iconographic information for children are other possibilities offered by this system. The information is conveyed to the passengers as relevant events during the trip. For example, such events may include approaching the bus stop at which the user wants to get off the bus. The architecture of the system is based on the Ambient Intelligence paradigm. The information needed by the users is located in the vehicles; users' applications access this information by using mobile devices (PDA, cellular phones) making use of local communication technology: IEE 802.15 (Bluetooth) and IEEE 802.11 (WiFi) that the vehicle's infrastructure makes available. The on-board infrastructure compiles information both from its own activity and from communication with the company's fixed infrastructure. Thus, the information system is distributed across the public transport network, including the vehicles themselves, and is delivered to the users locally. This is a major difference in comparison with other similar systems that apply a client-server model.

The system proposed is useful in two scenarios that imply different processes:

- On the one hand, the regular transport of passengers, where the users access the transport, pay for their tickets and are transferred to their destination The objective of this process is that the client is transferred to his or her final destination.
- The other scenario is for the transport of tourists. Although the process is equally simple, the aim is quite different; in this case the objective is that the client visit tourist attractions and obtain information about these attractions.

From a functional point of view, the system described is innovative in that it is an on route traveller's assistant that is not only capable of providing information about transport in real time (such as the next stop on the route, the estimated time of arrival at the next stop, arrival at the passenger's destination, estimated arrival time at that destination, among others), but it can also give tourist information about any point on the public transport vehicle's route in a format that is adapted to the passenger's preferences in terms of language and format (graphic or audio, *etc.*) From a technological perspective, the system includes the following innovative characteristics: it uses: (i) a distributed architecture, (ii) elements that are available in public transport vehicles (communications system and vehicle location system), and (iii) local communications infrastructure, specifically Bluetooth and WiFi. Moreover, the terminals that passengers need to access the information provided are mid-to-low range terminals; the only requisite being Bluetooth. This property of using local short-distance communication technologies, such as Bluetooth, has involved the resolution of some challenges, such as: service discovery, connection time and proper use of limited bandwidth that the traditional information systems for on route travelers must not resolve because they use mobile telephony infrastructure, such as 3G/UMTS.

This paper is structured in five sections. The following section is dedicated to explaining relevant related works in the field of on-route passenger transport information systems. The main technological challenges and innovative aspects of the system are presented in the third section. The system itself is described in the fourth section. The system validation tests are described in the fifth section. The last section is dedicated to presenting the main conclusions and future work.

A set of related works are found in the field of ITS architectures and frameworks for information services related to road transport, being two examples of this type EsayWay [4] and CVIS [5]. Both ITS initiatives make use of the basic technological infrastructures available on roads (sensors, vehicle-to-infrastructure (V2I) communications and traffic monitoring) in order to provide road transport information services. In the case of EasyWay, these information services are grouped into three areas: Traffic Management, Freight and Logistic Management and Traveller Information Services. In the case of CVIS, the services group are: Cooperative Urban Applications for improving the efficient use of the urban road network at both local junction and network level, and enhance individual mobility, Cooperative Inter-urban Applications for enabling cooperation and communication between the vehicle and the infrastructure on inter-urban highways, Cooperative Freight and Fleet for increasing the safety of dangerous goods transport and optimise transport companies' delivery logistics and Cooperative Monitoring for developing specifications and prototypes for the collection, integration and delivery of real-time information on vehicle movements as well as on the state of the road network. The main goals of the traveller information services of these ITS initiatives are the road transport safety improvement and the reduction of the traffic congestion and CO2 emissions. The information produced by these services are conceived to private transport user (drivers) and consist of real time warnings related to relevant incidents on the road, road signalling, weather forecasts, and travelling time predictions. This kind of information is implemented by short data messages and using mobile telephony technology (3G/UMTS).

The proposed system is a case of travel information system conceived for tourists travelling in public transport, providing interesting information, from a touristic point of view, adapted to the user preferences, for example language and media, and using the local communication infrastructure available in public transport vehicles, for example: Bluetooth and WiFi. Therefore, the following technological challenges has been faced: first, the amount of data associated to tourist information is potentially high, second the information can be accessed by several travellers of the same vehicle and using different user mobile devices, and finally, the use of local mobile communications implies the development of proper techniques for services discovering and connection establishment with a latency time assumable by the user. Conceptually, the proposed system could be considered as a case of traveller information service to be integrated in the infrastructure of the mentioned ITS frameworks.

## 2. Technological Challenges

As explained in the previous section, nowadays traveller information services are characterised by a centralised information structure; the users, using mobile telephony infrastructure, must connect to a remote server in order to gain access to the information. Generally, these services are based on Web Service technology and the user mobile devices must have advanced resources such as GPS. Another common property is that the information is related to relevant events occurring while travelling, this information interchange being implemented through short data messages. Important limitations of these services are: first, the functionalities for adapting to the user preferences are limited, and second, these services are conceived for private car drivers.

The system described in this paper is a system that provides tourist information in the context of a journey by public transport travel on the road. This information service has a high degree of accessibility for the users (tourist travellers). The information provided must be accessible through general-purpose mobile terminals that do not necessarily perform well. Therefore, access to the tourist information must be through local wireless communication networks that are available to these kinds of devices. The architecture of the proposed system meets the following requirements:

- Device heterogeneity. Tourist information should be available to a variety of general-purpose user mobile terminals.
- Interoperability. The system must be able to operate in the different technological and operation contexts of the public transport operator.
- Scalability. The system allows new elements to be added to the infrastructure that permit newly developed information services to be added or make them accessible to a greater number of users.
- Spontaneous interaction. The system allows the spontaneous interaction with users by the vehicle infrastructure; this number of users is potentially high.

The software system can be characterised by its capacity to integrate its surrounding physical and technological environment. Consequently, it can operate autonomously and spontaneously in different vehicles environments. To attain these functionalities, the system accepts that the number of users, devices and applications that intervene in an environment of public transport is unpredictable. A second principle accepted by the system is that the distinction between public transport environments must be made by boundaries that mark differences in content, and these boundaries do not need to limit the system interoperability. For this reason, a set of invariable operating principles that govern the execution of the system must be specified. Because of these characteristics, the system architecture is deployed in two areas. The first area is the infrastructure of the public transport network, specially the vehicle infrastructure. This includes a basic set of components, comprising all the elements that allow user applications to access tourist-related information. The second area is user devices, comprising all the components that have the capacity to integrate into the different vehicles environments and that facilitate access to the information produced by the tourist information services.

## 3. Related Works

Intelligent transport systems (ITS) aim to improve the safety, comfort and efficiency of both public and private transport. Advances in mobile communications have propitiated the development of infrastructure that enables communication between infrastructure and vehicles (I2V) as well as communication between vehicles (V2V) to take place, which has, in turn, led to the development of ITS including new services [6]. Giannopoulus [7] describes how the use of new information and communication technologies, especially those that are able to operate in any place or context, will change the way transport companies work, particularly in the case of the distribution of information to the traveller. According to this author, transport systems should be efficient, reliable and easy to use as well as highly adaptable to the needs and preferences of the users. In recent years different work has been carried out to identify the requirements that information systems for travellers must fulfil, especially travellers with special needs, such as the blind, deaf, or elderly. For example, Mitchell [8] and Waara [9] raise the information problems posed by elderly and disabled travellers using public transport and Jakubauskas [10] describes how smart transport systems can improve urban transport accessibility for passengers with reduced mobility.

On the implementation side, in the bibliography we can find several references to works that describe specific proposals of passenger information systems. In the field of information services for private transport passengers, services aimed at improving passenger safety using technological infrastructure based on intelligent sensors, mobile communication and location systems are particularly significant. This type of infrastructure is deployed in vehicles and on roads, giving rise to what are known in as smart roads. As examples, we could cite the Jang's proposal [11] of an environment for the development of on-board information services for the driver aimed at enhancing safety during the journey, and that of Pérez [12], who proposes a sensor system for infrastructure to vehicle (I2V) RFID communication that can transmit the information provided by active signals placed on the road to adapt the vehicle's speed and prevent collisions. The literature also includes references to the development of surveillance systems in public transport infrastructure to improve the public's safety in case of natural disasters of terrorist attacks. An example of this kind of systems can be seen in Proto's work [13], which describes a surveillance and monitoring system for large transport infrastructures (airports, transit stations, motorways, *etc.*) based on a network of local sensors and others distributed in the infrastructure of large transport networks.

The technology available in vehicles varies depending on the kind of vehicle and the type of services to be provided. However, international standards covering on-board architectures have already been put in place, an example of which can be seen in ISO/DTR 13185-1.3 [14].

In our opinion, systems conceived for people with special needs are especially interesting. In this vein, we find the work of Turunen [15], who describes a system that provides audio information to guide blind people in an intermodal transport context, where the user accesses the services through his mobile device (PDA or mobile telephone). Sanchez [16] describes the AudioTransantiago system, an information system that facilitates access to the Santiago de Chile public transport network to blind people. Barbeau [17] describes a system named Travel Assistant Device (TAD) that gives special needs users equipped with a mobile telephone with GPS, information about their trips, specifically for users who have planned their journey in advance. The system guides them by providing information in real time as to their position, warning them if they deviate from their route and notifying them when they have to ask for the bus to stop. In the specific case of information systems for tourists, we highlight the case of the system proposed by O'Grady [18], which provides information on the route, guiding a traveller from one point in the city to another on foot. Finally, in the field of systems inspired by the ubiquitous computing paradigm, Arikava [19] proposes a multi-modal transport information system. All these systems share a common architectonic characteristic: they have a client-server architecture where the information is located in a central server and the clients access the services by using Web technology from their mobile devices. The system described in this paper has a distributed client-server architecture, so the Server is executed in the vehicle in which the passenger is travelling and the communication between this Server and the client application that is executed in the mobile device takes place thanks to the local communications infrastructure available in the vehicle (Bluetooth).

## 4. Description of System

The objective of our system is to develop an infrastructure for the distribution of multimedia information to mobile devices. This system must be able to provide contents (audio, video, text) to the travellers as the vehicle travels past certain relevant points on the route.

It adopts the goals of traditional public road transport information systems for passengers. These kinds of systems provide passengers with static information, such as departure timetables and estimated times between stops, fares, *etc.*, or dynamic information, such as anticipated times of arrival of vehicles, changes in circumstances, delays, *etc.* These information services can normally be accessed on Internet pages or are provided in panels placed in stations or at stops.

The proposed system provides the information directly to the user by means of personal mobile devices, mainly cellular telephones, which are commonly used nowadays. The direct interaction between the user mobile devices and the public transport infrastructure, basically the transport companies' production systems, is carried out in such a way as to offer the user information immediately, and it is this type of interaction constitutes the system's most noteworthy characteristic.

In this sense we have used the philosophy of the ubiquitous computing and ambient intelligent (or AmI) paradigms, trying to integrate technological elements into the users' daily life in a transparent way that requires the smallest possible adaptation effort on the part of the users. These technological elements are already in their hands and our job is to maximise the advantages they offer as Fuentes [20] proposes when he explains the three properties that the AmI devices must fulfil, benefiting both the user with enhanced facility, flexibility and reliability in information access, and the transport companies, who benefit not only from improved information distribution, but also from the external resources provided by the users. For this reason, the system constitutes an innovative tourist information system: during a public transport vehicle's journey, the system can offer information concerning points of interest, such as cathedrals, monuments, shops, buildings, *etc.* by audio, video or text contents, to the travellers.

In the system developed there are three major elements: the Client Application, the on-board Information Server and a repository from whence the user can obtain the Client Application. The Client Application runs on the user's mobile terminal, the on-board Information Server executes on a computer that forms part of the infrastructure of the vehicle (bus) and the repository is located in a Web site. From the repository, the user can also download information supplied by the company about different documented routes. Each documented route is represented by a package with multimedia files that will be used by the Client Application during its normal operation. A user must obtain the Client Application and the package that contains the data files for each route he is interested in. During the trip, the Client Application communicates with the bus's on-board computer in order to get all the data required to assist the traveller. This is done using the on-board Bluetooth communication infrastructure because this is the technology most frequently supported by user mobile devices. Another reason for using this technology is to avoid the communications infrastructure used by the production processes that run on the vehicle, such as WiFi. The information service should interfere as little as possible with the production processes running on the transport infrastructure and it must use the infrastructure resources as little as possible.

From the perspective of system users, we can distinguish four main roles:

- **Administrator or content manager:** the user is in charge of managing the contents in the repository. It uses the application installed in the central station and its function is to load the contents and associated position tables.
- **Activator of the service:** in public transport, this role may not exist as the activation of the service can be carried out automatically using the infrastructure available in public transport vehicles. By contrast, in discretional passenger transport there has to be a person in charge of activating the service in the on-board Information Server at the beginning of the journey. The best person to carry out this action is the vehicle driver, although this action should not affect his/her main function of driving the vehicle.
- **Clients:** these will be the users of the application to be installed in mobile terminals. Apart from permitting the installation of the application in their devices, a basic initial configuration will also be required.
- **Contents generator:** although this user does not form part of any of the elements of the system implemented, it plays an important role. In order to access all the information available about a route, the information must be studied carefully. To this end, it is necessary:
  - To compile points of interest.
  - To obtain their coordinates using a GPS receiver.
  - To carry out a study of the most relevant aspects of the point of interest.
  - To generate the information.
  - To generate the associated audio file.

Once this process has been carried out, we will be in a position to include these new contents in the repository so that the clients can download them. Currently, the online distribution of multimedia data is not available, because the prototype developed uses Bluetooth and the distribution of multimedia information by means of this technology is too slow. Therefore, and bearing in mind that the goal is to synchronize the delivery of information with the passing of the vehicle through a sequence of places of interest during the route, we thought was more feasible that the user download the files in advance, so that the Information Server sends a message to the client application signalling which file to reproduce and the exact moment the reproduction should be initiated.

### 4.1. Vehicle Infrastructure

**Figure 1.**
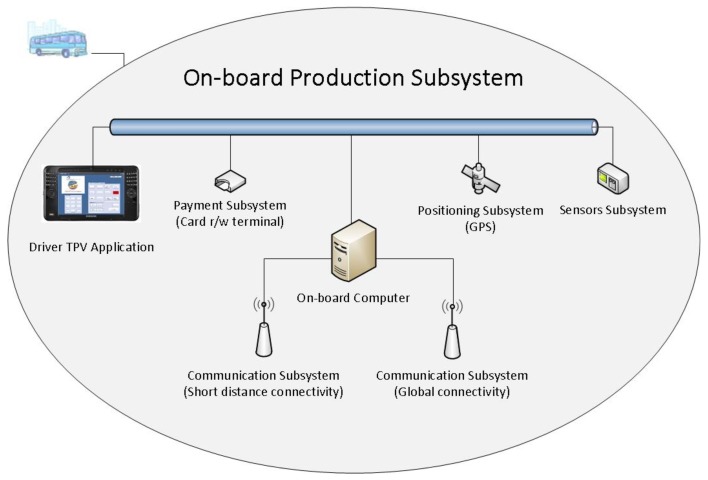
On-board production subsystem.

The system assumes that vehicles are equipped with all the elements required for them to be able to control their activity autonomously. In the context of public transport, this means that the vehicle infrastructure has all the resources needed to perform tasks related to the control of payment and planning without a permanent connection to a control centre. From a functional point of view, these elements can be grouped as follows (Figure 1):

- **On-board computer:** with the computing, storage and communication resources required to execute the process related to production activities. In our case, we have an embedded computer configured by a low-power processor, 64 Mbytes of main memory, solid state disk of 1 Gbyte, serial communications interfaces (RS-232/485), network interface IEEE 802.11 and a Bluetooth interface.
- **Positioning subsystem:** this is configured by all the elements providing information to the system as to the vehicle's location. In our case this subsystem is formed by a GPS receptor.
- **Communication subsystem:** for transmitting and receiving information (voice and data). Long distance communications are supported by public infrastructure (radio, mobile telephones) or by private infrastructure (normally radio systems). In our case, a trunking radio public infrastructure is used; the data travels in short packets of data associated to relevant events. A wireless local net (IEEE 802.11) is used to transmit large amounts of data between on-board systems and the company's information system.
- **Payment subsystem:** configured by the elements required for on-board payment, normally: a driver console and contact-free card terminal.
- **Sensors subsystem:** these elements enable the on-board system to access critical parameters related to the safety of the vehicle (for example, the open doors alarm), electrical parameters (for example, the battery voltage level) or environment (for example, temperature).

The elements of this infrastructure used by the system are: the on board computer, the positioning subsystem and the communications subsystem.

### 4.2. Conceptual Data Model

Interoperability is an important characteristic of efficient public transport. Interoperability means the capacity to integrate applications developed by different suppliers. This integration enables the exchange of data between different software products to take place. For this reason, operators and authorities are interested in using standard specifications to facilitate this integration. A conceptual data model specification exists in Europe. This model, named TransModel [21], includes ontology, a set of entities and relationships about basic data needed to describe the network, the handling of the different data versions and the information needed for different domains of public transport, including: tactical planning (vehicle scheduling, driver scheduling, rostering), driver disposition, operations monitoring and control, passenger information, fare collection and management information and statistics. The system uses this conceptual specification including data descriptions that go far beyond the planned timetable, which is the main source of traditional timetable information, but does not take into account any dynamic issues. Specifically data concepts of this specification refer to passenger information facilities, conceptual components of a passenger trip, definitions needed to calculate trip duration, the times at which individual stops are passed on journeys and service modifications that are consequences of exceptions to the original plan. Basically, the system uses elements of the topological network definition (lines and journeys), geographical information and information regarding specific types of passenger. From the point of view of the formal context representation for ubiquitous contexts, said representation follows the specifications introduced by Hervas [22]; these specifications identify the initial requirements to model the context.

### 4.3. Client Application

**Figure 2.**
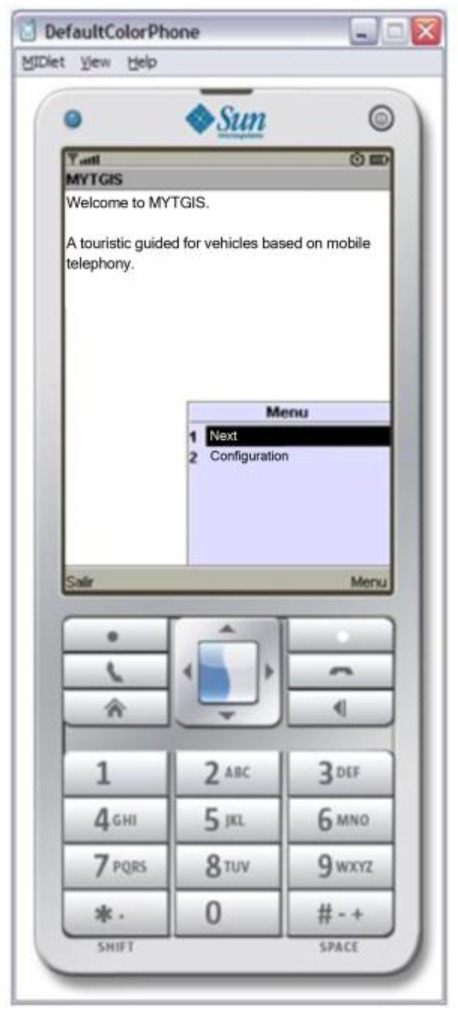
Client application screen executing on a mobile phone.

In order to deal with the heterogeneity of users' mobile devices of, the Client Application has been developed in the form of a Midlet, so each mobile device is required to implement JavaME (Figure 2). This election has been made because, in our opinion, it is a way of guaranteeing that this application provided by the system can be executed in a large number of mid-range terminals and the adaptation enabling it to run on other types of terminals, such as Android terminals, is relatively easy. The heterogeneity of mobile devices in the market also affects the way in which data are stored and organized in the devices, making it impossible to take a common file system structure for granted; consequently, static data required by the application are embedded in the Java application itself. The first step to be performed in the Client Application is its configuration. In order to achieve the configuration, the user has to select the communication technology to be used—only in terminals with alternative communication technologies, such as Bluetooth and IEEE 802.11. When the user begins a guided trip, he must specify his preferences: define the bus route (trip) that he wants to take, his destination and how he wants to be informed. At the moment the system has two modes of reporting information:

- **Arrival alert.** In this mode, the passenger is notified at the arrival to the destination specified in advance. The warnings are issued three times: when the vehicle is at the bus stop before the one selected, the second warning is given just before arrival and the third warning is issued when the vehicle stops at the destination bus stop.
- **Detailed guidance.** In this mode, the user is notified at each point of interest that the vehicle comes to during its journey. The points of interest are selected following a criterion determined according to the intention for which the system is programmed: tourist, urban information, cultural places, administrative centres, *etc.*

As far as the graphic interface designed by Client Application is concerned, as this is an application for mobile devices, a simple interface that is easy to use has been chosen. Its simplicity is based on the following factors:

- Limitations in the features of the mobile devices. Although the latest generation devices have improved considerably, they still suffer important restrictions in terms of screen size and data processing capacities.
- The wide variety of target users makes it important to ensure that the application is user friendly.
- Lastly, the success of any application depends on how easy it is to use.

**Figure 3.**
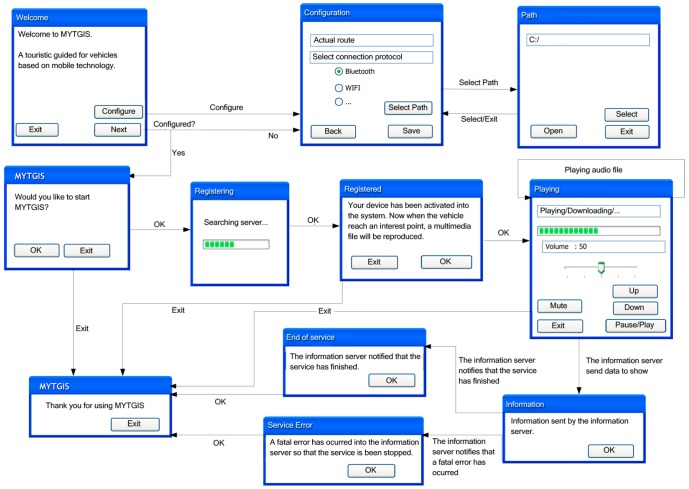
Flow diagram of the Client Application.

Figure 3 shows that the execution flow from the Client Application and the different forms of interaction with the user.

The first screen shows three options:

- “Exit”, which terminates the execution, showing the disconnection form.
- “Configure”, which facilitates access to the configuration screen. From the configuration screen, when we press the buttons “Save” or “Cancel”, we access the first screen once again. Moreover, it is also possible to access the forms to select the work route by pressing the “Select Route” button. In this form, there are three options: “Open”, which opens the file selected to continue with the operation; “Select”, which allows you to select the configuration folder, and “Exit”, which takes you back to the configuration screen.
- “Next”, which allows you to move forward in two different directions: to the configuration screen, if configuration has not been effected, or to the initial form to initiate registration in the server, if configuration has already taken place. Once acceptance has been given for registration to take place, a screen comes up indicating that this process is underway, and when it is completed, the registration confirmation screen comes up, informing the user that the device is ready to start to reproduce the contents once a place of interest has been reached. From this screen the application can also be terminated by selecting “Exit”, in which case the service is asked to terminate the client's registration.

The reproduction screen presents various options:

- Volume Configuration: up, down or silence.
- Reproduction options: pause or continue.
- Exit, which terminates the execution of the client's application.
- Once the Client Application is in “reproduction” mode, depending on the information provided by the server, several information screens can come up automatically: a screen with information sent by the server to the client; a screen informing that the service has come to an end or a screen informing that an error has taken place. In the last two cases, the execution of the Client Application will terminate.

**Figure 4.**
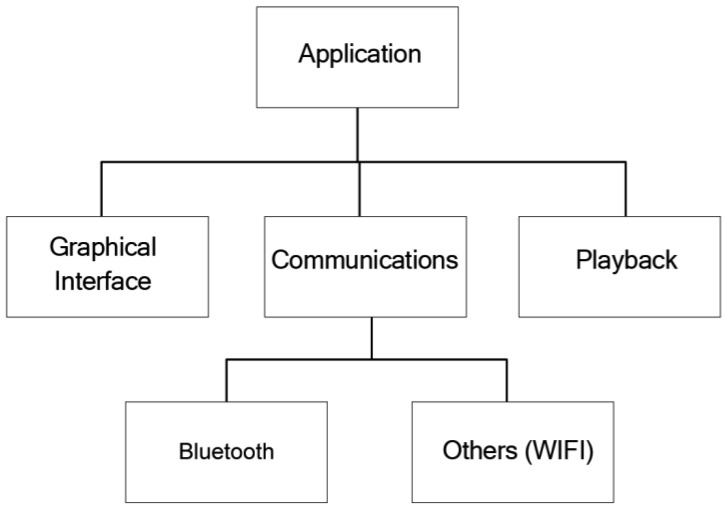
Modular structure of the Client Application.

From the point of view of the design of the Client Application, three differentiated modules can be appreciated (Figure 4): the graphic interface, communications subsystem and contents reproduction subsystem. The design pattern used is the Model-View-Controller pattern. The communications subsystem is made up of a main module that acts as an interface with specific communication types, thereby enabling modifications to be made in the underlying layer of communication without having to affect the rest of the application. Currently, only communication via Bluetooth is implemented.

**Figure 5.**
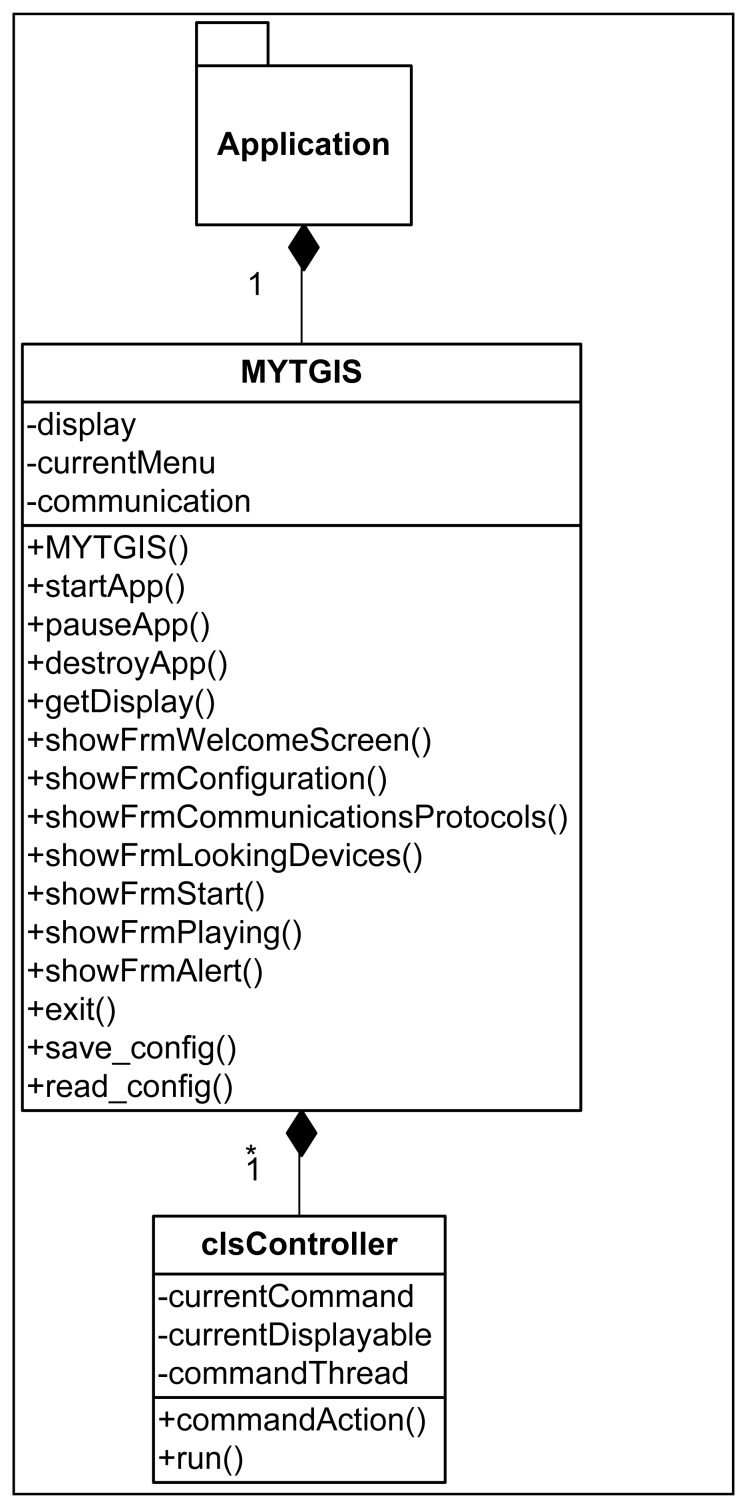
Class diagram of the graphic interface.

Figure 5 shows the class diagram where we can see the attributes and methods of the classes that make up the graphic interface of the Client Application.

**Figure 6.**
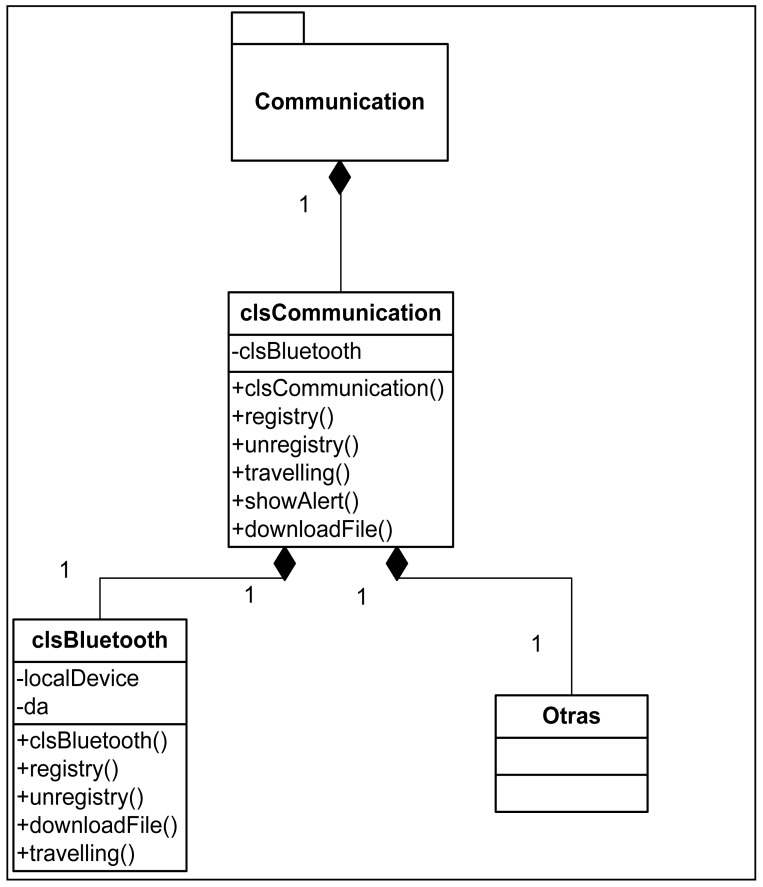
Class diagram of the communications subsystem.

**Figure 7.**
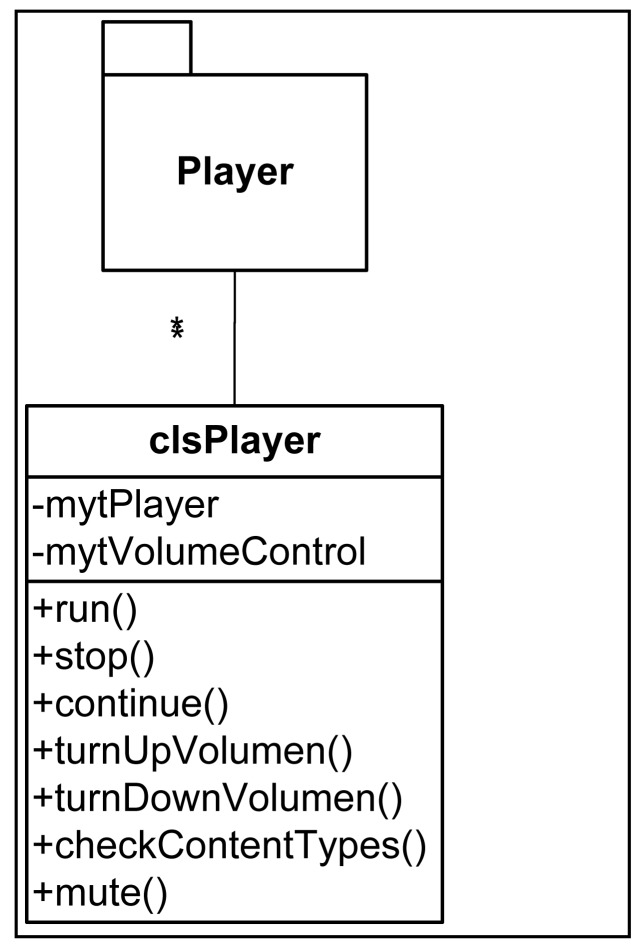
Class diagram of contents reproduction subsystem.

The communications system has been designed based on a clsCommunication type that manages the communications regardless of the protocol used (Bluetooth, WIFI, *etc.*). The class diagram of this subsystem can be seen in Figure 6. Finally, and to round off the Client Application design, Figure 7 shows the class diagram of the contents reproduction subsystem.

Now that the Client Application modules and design have been explained we can explain how it is executed. It must be remembered that the main objective of the Client Application is to provide information of interest to the client, reproducing appropriate sound files depending on the specific point of the route at which the vehicle is located.

Initially, the user has to choose the communication infrastructure to be used: Bluetooth, WIFI, or others. Subsequently, the Client Application must automatically carry out the steps required to activate the necessary services that allow it to use the infrastructure chosen. It then starts the search among other devices that offer the service chosen. Once the on-board Information Server has been identified, the connection is established. In order to facilitate subsequent connections established by the server with the client, in this first connection, once the server has been selected, the client sends the necessary information so that the server knows that the device is using its application; this means that there is no need to repeat the search process for a device each time it sends a new communication to clients. When a device stops using the application, the server is informed and said device is eliminated from the list of devices connected. Once it has registered, the Client Application waits until the server sends it a file to be reproduced. During reproduction, the Client Application continues to wait for new communications. Finally, if the client terminates the application, this event is communicated to the server and it eliminates said device from the list of devices connected.

The structure of the data packets used in client-server communication is simple. It is formed by two fields: the first is called Information Type, which is an integer value that identifies the type of information so that the client knows what to do with the information. The second field is called Information, and it consists of a string of characters that, depending on the Information Type field, may be a command, information to be shown or information about the service. The “#” character is used to separate fields. Table 1 gives definitions for the types of information and possible values and meaning of the information field in each case.

### 4.4. The Information Server

The Information Server is installed in the vehicle infrastructure. Its task is to receive subscription requests to the service from clients and to send the corresponding messages to them when the vehicle comes near to a point of interest. The service will begin to operate after a command sent by the vehicle to start the Server. The Server will not require much interaction with the vehicle infrastructure.

**Figure 8.**
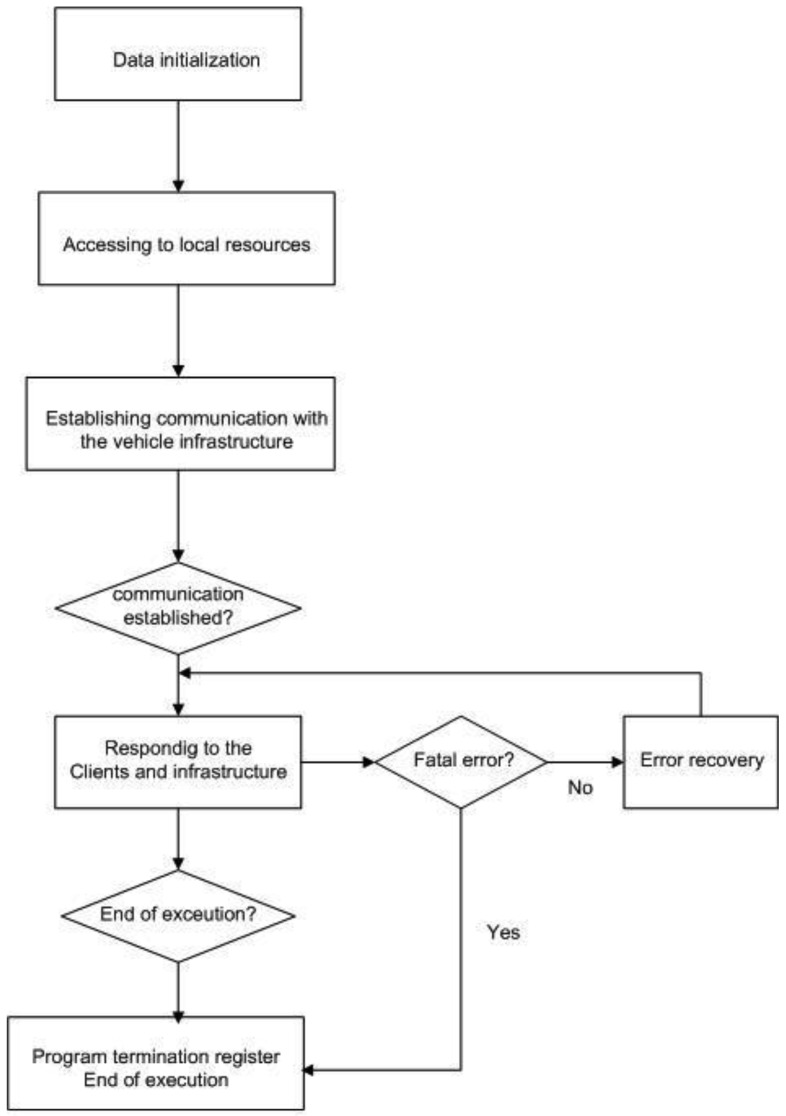
Flow diagram of the information server.

The working of the on-board Server can be described as a machine that goes through the following stages (Figure 8):

- Start-up (S0). At this stage, all the control structures are initiated, all checks are made and connection with the on-board infrastructure is attempted.
- Normal working stage (S1). This stage can be reached from S0 and S2. It is reached from S0 when all previous checks have been carried out and connection is successfully made with the infrastructure. It is reached from S2 when the anomaly detected has been overcome and communication is operative with the infrastructure and the user applications that have previously been registered.
- Error stage (S2). This stage is reached from S1 when an error has been detected. The error in question may be internal to the server application itself or external, such as a loss of connection with the on-board infrastructure. If the error can be corrected, stage S1 is returned to. Otherwise, S3 is reached, or stage S2 will be maintained until the error has been resolved, for example if communication with the infrastructure s lost, it will wait until communication is re-established.
- End stage (S3). This stage is reached from stage S1, as a result either of a request by the infrastructure to terminate execution or of a serious error.

We will now describe the set of datagrams that travel between the vehicle's infrastructure and the server, thereby giving regular information as to state of the vehicle's infrastructure. Firstly, the fields that make up the different datagrams are described in Table 2, and then the datagrams used are listed and, lastly, a description of each is given.

The different datagrams are listed in Table 3, together with a description and the different states of the server application in which the datagrams in question are used. These datagrams basically describe the situations that need to be communicated to the server by the vehicle's infrastructure.

Let us know look at how each datagram is structured and its use.

The datagram showed in Table 4 is used to indicate that the vehicle's infrastructure is in error mode and consequently the Server needs to communicate to the clients the fact that the service is not available.

The datagram represented in Table 5 informs us that the vehicle has gone into “not in service” mode. No service has been started so the Server cannot attend any requests.

The datagram described in Table 6 indicates that the vehicle is on an “Operational Stop”, *i.e.*, in transition between two journeys. We do not yet know what the next service the vehicle will offer will be so the server is unable to receive requests.

The datagram showed in Table 7 gives information about the starting up of a vehicle's service. It marks the beginning of the service. As of this moment, the Server has to admit subscriptions because it has the necessary information.

The datagram described in Table 8 is the most important because it gives information as to the arrival at each stop.

The datagram showed in Table 9 indicates that the vehicle has had to stop the service for some reason. The passengers may have had to change vehicle. In principle, this would mean that the service would terminate.

The points of interest are compiled in a data base that associates a GPS position with an attribute that can be textual information to send to the client or the description of a file that the client has to reproduce. In both cases this information is sent to clients who have subscribed. In terms of the GPS location of a point of interest, a precision correction must be incorporated by setting a margin of error around the point in question. The size of this margin will be defined according to geographical circumstances.

**Figure 9.**
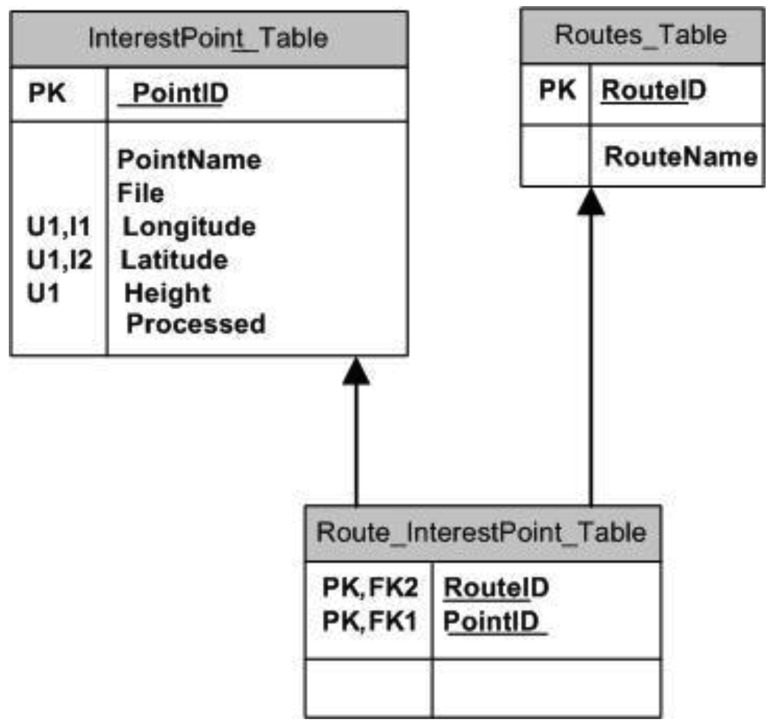
On-board data base with tourist information.

From the point of view of the data, content management is supported by a data base comprising three tables: a table of routes (Routes_Table), one including points of interest (InterestPoint_Table) and the third that links routes and points of interest (Routes_InterestPoint_Table). The on-board data base is represented in Figure 9; in this figure the diagram shows the relationship between the tables:

The routes table contains a set of lines that make up the transport company's network. It contains two fields: the route number (RouteID) and its name (RouteName).

The table giving points of interest is very important, as it holds most of the information that the service will process and communicate to clients. It is made up of the following fields:

- PointID: An integer-type field that uniquely identifies the point. This will also be the table's primary key (PK).
- PointName: Representative name of the point of interest. A string-type field.
- File: A string with a maximum size of 255 characters. It indicates the file to be reproduced that is linked to the particular point of interest and is therefore what clients have to download. It will be made up solely of the name of the file and as no two files with the same name may co-exist, it must be the only one stored in its folder with that name.
- Longitude: decimal-type field representing the Longitude of the coordinate at which the point is located. It will be expressed in degrees and decimals.
- Latitude: decimal-type field representing the Latitude of the coordinate at which the point is located. It will be expressed in degrees and decimals.
- Height: Another decimal-type field, this time representing the height in metres (above sea level) at which the vehicle is located.
- Processed: This Field indicates whether or not the point has previously been chosen from a different position. This field is necessary as a point of interest appears when the vehicle comes into a radius of proximity, so it is quite likely that in subsequent consultations with the data base, several coordinates fulfil the conditions pertaining to the same point of interest.

The coordinate has been separated into three fields to facilitate storage, on the one hand, and, more importantly, data retrieval. Moreover, in order to make the search process easier, two indices I1 and I2 have been created. The first is an index for the Longitude field while the second corresponds to Latitude. No index is used for the height field for two reasons: firstly, it would not enhance the searches, and secondly, because it would penalise data input and elimination. In any case, this will not have any serious consequences because this type of operation is not very common and tends only to take place at the beginning of the software installation. At the same time, a restriction of uniqueness has been established for the coordinate but said restriction has not been established on the field that stores the name of the associated file (Field File) as it could be the case that two different coordinates had the same associated file. It is also semantically correct because there may be two routes that go past the same point of interest but at different coordinates, for example if one goes past the front of a cathedral and another passes the back end.

Given that several routes may coincide in one or more points of interest, the routes must be linked to their points of interest. This linkage is reflected in the Routes_InterestPoint_Table table that contains two fields: Route Identifier (RouteID) and the point of interest identifier (PointID). This table, apart from the primary key made up of both fields, has two foreign keys that link the RouteID and PointID Fields, with the Route and Points of Interest tables, respectively.

This data base must be complemented with the set of audio files that can be reproduced.

**Figure 10.**
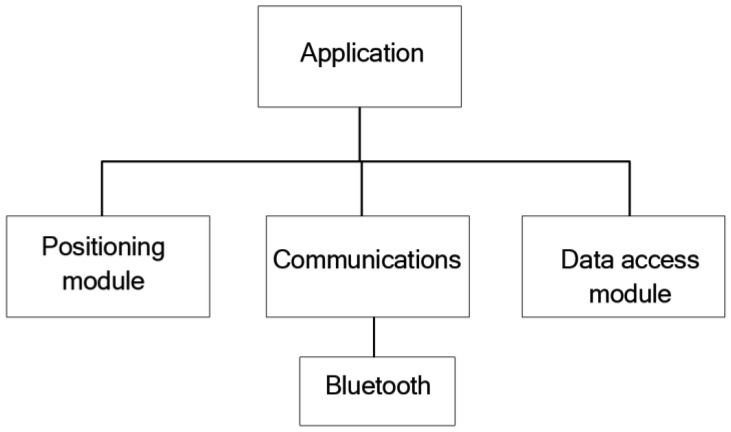
Modular structure of the Information server.

From a design point of view, the Information server comprises three modules (Figure 10):

- Positioning module: this module is in charge of obtaining the coordinates of the positioning system and to check if its current position includes points of interest for tourists. To this end, it has to access the database, so it needs to use the data access module.
- Communications module: this module manages the service that is published and offered to clients. It also manages the incoming communications requesting registration and the outgoing communications requesting files.
- Data Access module: this module facilitates all those methods required to manage the data base data.

**Figure 11.**
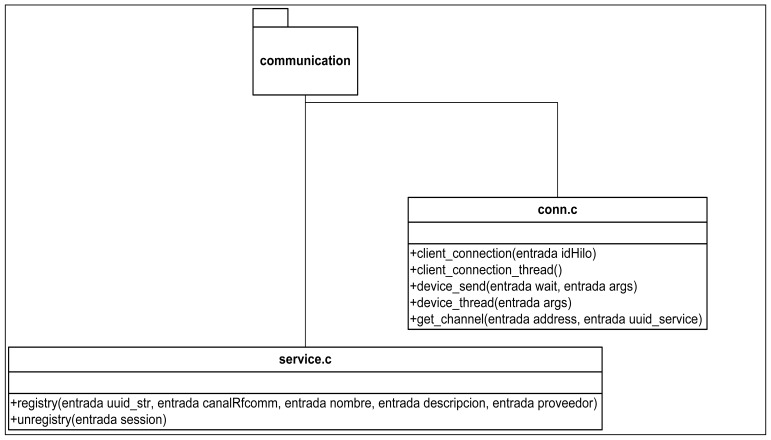
Class Diagram in the communication subsystem.

**Figure 12.**
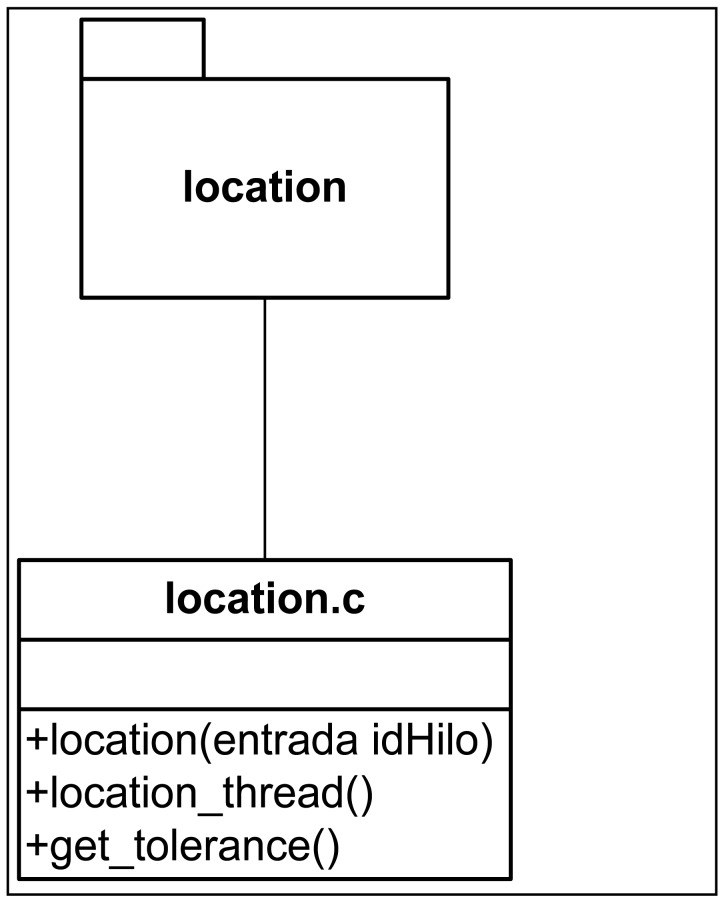
Class Diagram in the positioning subsystem.

**Figure 13.**
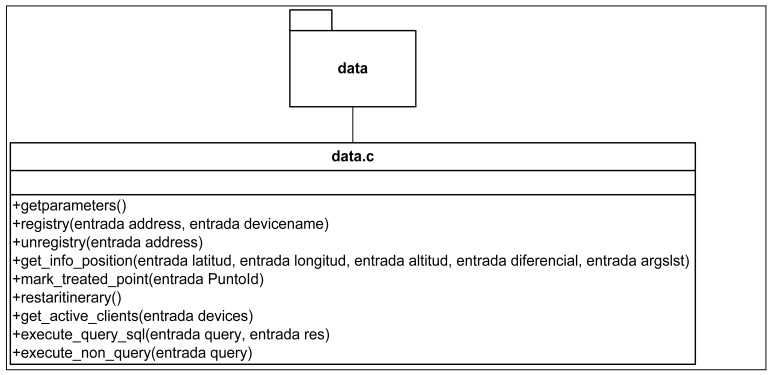
Class Diagram of the data access subsystem.

Figures 11–13 show the class diagrams of each of the modules that make up the Information server.

The Server functionalities are achieved by three simultaneous tasks performed by the execution of three concurrent threads:

- **Registering:** This task waits for devices requesting subscription to the service. It creates and keeps the list of clients and their particular requirements. It erases from the list the devices that have reached their destination.
- **Location:** This task is permanently asking the infrastructure of the vehicle for its location. With this information it checks whether the vehicle is in the neighbourhood of a point of interest.
- **Warning messages:** When the location task detects that the vehicle is at a point of interest, this task must inform all interested clients with a message containing explicit text about the point of interest or instructions to the client application telling it to reproduce some of the multimedia files associated with the route in question. There will be a thread of this task for each subscribed client because all interested clients must be notified at the same time. Figure 3 shows the execution flow of the Information Sever.

Now that we have explained the different modules and design of the Information server, we will go on to describe the execution flow in the said application. Once the service has been initiated and Bluetooth activated, the registration of the MYTGIS service takes place in the Service Discovery Protocol server. The localization and management of registration threads are then created; these threads will remain active throughout the execution of the service. From this point on, therefore, there are three concurrent execution flows, each associated to the three threads created.

The localization thread takes care of checking the current position and checking relevant client information. The registration management thread is ready to receive client requests to carry out a registration or disconnection operation.

In the thread that manages communication with the devices, the following flow of actions occurs: first of all, the device is put on hold for clients who request use of the application; when this happens, the new device is registered and included in the system as a registered device. This thread also takes care of requests received from devices wishing to abandon the system.

The localization thread constantly checks to see if the vehicle's current position has changed. Each time it moves, the thread carries out a check in the database to see if there is any point that coincides with the current location, which would mean that it is drawing close to a point of interest. At this point in the process, the set of active devices is consulted and a thread created for each of them, to communicate the new information to be presented, in the selected format, to the user.

The main thread delegates the work to the threads described, apart from the user command interface, which enables notifications to be sent regarding a change in location, the sending of information or commands to clients. The only command that leads to the main thread terminating its execution is the “Exit” command.

## 5. System Validation Tests

In this section, the tests carried out to validate the system developed are described. Given the aim of the system and its architecture, the tests have focused on analyzing two key aspects that affect the validity of the system: local communication between the Information Server and the Client Application and the reliability of the information provided to the passenger.

The validation tests were carried out in the laboratory using a simulation environment. In order to ensure that this simulation environment reproduces real situations that occur in a public transport company, we have collaborated with the public passenger transport company Global Salcai-Utinsa of the island of Gran Canaria. This company allowed us to install in the on-board systems of a set of vehicles belonging to their fleet a programme that continuously recorded data regarding: (a) the GPS location of the vehicle (latitude, longitude, height, speed, date and time of the measurements taken and quality of the measurement), and (b) the events that took place in the vehicle (starting off on the routes, end of routes, passing stops, passengers boarding and disembarking and technical alerts). This programme obtained the information described by periodically accessing the vehicle's on-board computer. Using the average speed of the vehicle as a criterion, these routes were classified into three types: fast routes, intermediate routes and slow routes. The reason for taking the vehicle's speed as a criterion is because this parameter allows us to establish different system response time requirements when passing a point of interest. Once a route belonging to each of the three categories had been chosen, the recording programme was run for a period of four months in the vehicles that operated the chosen routes. The recording programme organised the data obtained in three files: one for location, one for events related to the routes monitored and the last for technical alerts. These three files were periodically transmitted by the vehicle's mobile communications system to the laboratory and were processed in order to incorporate the data into the simulation system's databases. The laboratory simulation consisted of executing a programme that interrogated the data base in order to generate the information provided (locations and events on a route over a period of time) as input data for the simulator. Thus, the tourist information system developed could be tested using data representing real situations that had occurred in the vehicles.

The mobile devices used during the tests were mid-to-low range mobile telephones. Specifically, mobiles with the Symbian operating system were used, with the only requirement being that they had Bluetooth for wireless communication.

With this set-up in the laboratory, the tests were carried out simulating two scenarios. In the first, the system was validated during a route; this scenario is useful for the validation of the system's response times, which have to be appropriate bearing in mind the different speeds at which the vehicles go past the various points of interest. The second scenario aimed to simulate the situation in a bus station, where a number of different information systems are available and the client connects to a specific system. This second scenario is useful for the validation of the different times needed by the client applications to detect the information service and subscribe to it.

As Bluetooth technology was used, the tests used in the analysis of local communications have been developed to obtain two key times: the time it takes for the Client Application to discover the service, once the connection time with the Information Server has been discovered, and secondly, the time it takes to transfer the data that trigger the multimedia reproduction of contents by the Client Application. In order to analyze the implications of the number of Bluetooth devices using the services, a variable number of Bluetooth devices participated in the tests, specifically from 2 to 8, which is the maximum number that a Bluetooth network can manage with just one Bluetooth station server (piconet). The tests have determined that the time it takes to discover the service varies between 10 and 20 seconds depending on the number of Bluetooth devices connected to the network. Once the service has been discovered, the connection time is short in comparison with the previous time and it varies very little, between 3 and 5 seconds, regardless of the number of Bluetooth devices connected to the network. The transfer time necessarily depends on the amount of data to be transferred; specifically transfers of 1 Kbyte took about 2 seconds, 79 Kbytes, about 15 seconds and 300 Kbytes around 2 minutes.

Response time was used to measure the level of reliability of the information provided to the passenger. In this context, response time is defined as the time that passes from the moment a relevant event to be communicated to the passenger occurs, such as the vehicle passing a point of interest on its route, until the event is communicated using the preferences established by the passenger in his/her Client Application; this could be, for example, a voice message or a text message. The tests carried out have consisted of communicating different types of events with response time restrictions and different amounts of data to be transmitted. In terms of the transfer terms described above and the different lengths of time taken by the vehicle to go from one interest point (for example a stop) to the next, it was decided to limit the amount of data transmitted to communicate an event to a maximum of 1 Kbyte.

## 6. Conclusions

The development of information and communications technologies, especially in contexts of mobility, has allowed us to incorporate new functionalities into the information system for public transport and specifically into passenger information services. A special case of this kind of system has been described in this paper. This system is based on the ubiquitous and ambient intelligence paradigms and the main goal it achieves is that of facilitating access to public transport and providing information of interest to tourists. Unlike on-route public transport passenger assistants, this system is capable of informing about any point of interest on the route taken by the vehicle, adapting the information to the passenger's preferences, such as language and information type (audio, graphic or text), all of which are supported by the system.

The structure of the system comprises three main elements: the Client Application, the on-board Information Server and a repository from which the user can obtain the Client Application. The Client Application runs on the mobile terminal of the user, the on-board Information Server executes on a computer that is a part of the vehicle's infrastructure and the repository is located in a Web site that is freely accessible by users. From a technological point of view, the system presents the following innovative characteristics: firstly, its structure is distributed, which means that the tourist information servers are deployed in the fleet of public transport vehicles and not in just one server; secondly, as a consequence of the first characteristic, passengers access the information using local communication infrastructure and those available in the vehicles, specifically in this case Bluetooth as communication technology and GPS; and lastly, the passengers' mobiles needed in order to access the information are medium-to-low range, the kind of devices that are currently very popular.

In order to improve the system and as future work several lines of research must be developed. One is the use of Android technology to develop the Client Application. A second line is to improve the system architecture introducing a new element, Service Broker, to facilitate the information service searches executed by the Client Applications, and finally, the use of ZigBee technology for communications with the passengers' mobile terminals.

## Figures and Tables

**Table 1.** Types of information and values used in the data packets

**Information Type**	**Information**	**Meaning/Action**
0	gen_error	Command indicating to the Client that a Server error has occurred and that execution will terminate, so the Client should also terminate.
0	gen_exit	Command indicating to the client that he should terminate execution.
0	gen_stop	Command indicating to the client that he should terminate the reproduction underway.
1	file_name	Indicates to the client that he should reproduce the file appearing in the Information field.
2	Information appearing	Server message indicating that a message must be shown to the client with the information given in the field of the same name. This may be useful, for example, for indicating stops.

**Table 2.** Description of the fields used by the different datagrams.

**Field name**	**Length in bytes**	**Description**
CT	1	Start of datagram. Fixed value of 0xFF
TT	1	Specific type of datagram: 0 Datagram indicating that the service is off-line. 1 Datagram indicating that the system is trying to re-establish following an error. 2 Datagram informing that the vehicle is out of service. 3 Datagram describing the attributes of a line. 4 Datagram describing where on its journey the vehicle is currently located. 5 Indicates that the vehicle is on an “Operational Stop”. 6 Datagram indicating a pause on the line. 7 Datagram informing that an order to stop has been received from the infrastructure.
CL	6	Line number
NL	50	Line name
SL	1	Direction: 0 Outward journey 1 Return
CP	10	Stop code
NP	20	Stop name
LL	3	Number of stops on line
CV	6	Vehicle number
NS	3	Sequence number

**Table 3.** List of datagrams.

**Datagram identifier**	**Stages of the server application at which it is used**	**Description**
1	E0, E1, E2	Indicates that an error has been detected in the Server application and that attempts are being made to solve the problem
2	E1	Indicates that the vehicle is not in service
3	E1	Indicates that that vehicle is on an “Operational Stop”
4	E1	Describes the attributes of a line to be followed
5	E1	Describes where on the line the vehicle is
6	E0, E1, E2	Forcible termination of service

**Table 4.** Datagram T1.

**Field**	**Length in bytes**	**Meaning**
CT	1	Start of packet, fixed value of 0xFF
TT	1	Fixed value of 1
CV	6	Vehicle number

**Table 5.** Datagram T2.

**Field**	**Length in bytes**	**Meaning**
CT	1	Start of packet, fixed value of 0xFF
TT	1	Fixed value of 2
CV	6	Vehicle number

**Table 6.** Datagram T3.

**Field**	**Length in bytes**	**Meaning**
CT	1	Start of packet, fixed value of 0xFF
TT	1	Fixed value of 3
CV	6	Vehicle number

**Table 7.** Datagram T4.

**Field**	**Length in bytes**	**Meaning**
CT	1	Start of packet, fixed value of 0xFF
TT	1	Fixed value of 3
CV	6	Vehicle number
NS	3	Sequence number
LL	3	Number of stops on the line
CL	6	Line number
NL	50	Line name
SL	1	Line direction
CP	6	Code of first stop
NP	20	Name of first stop
CP	6	Code of stop i
NP	20	Name of stop i
CP	6	Code of last stop
NP	20	Name of first stop

**Table 8.** Datagram T5.

**Field**	**Length in bytes**	**Meaning**
TX	1	Start of packet, fixed value of 0xFF
TT	1	0 if on the move or 1 if in pause mode
LL	3	Number of stops on the line
CL	6	Line number
SL	1	Direction
NL	50	Line name
5	10	Number of the last stop passed
6	25	Name of stop
7	10	Number of the next stop to be passed
8	25	Name
9	6	Time in seconds remaining before reaching the next stop

**Table 9.** Datagram T6.

**Field**	**Length in bytes**	**Meaning**
CT	1	Start of packet, fixed value of 0xFF
TT	1	fixed value of 6
CV	6	Vehicle number
